# *KaiBiLi*: gesture-based immersive virtual reality ceremony for traditional Chinese cultural activities

**DOI:** 10.1186/s42492-025-00205-x

**Published:** 2025-10-02

**Authors:** Yiping Wu, Yue Li, Eugene Ch’ng, Jiaxin Gao, Tao Hong

**Affiliations:** 1 School of Mathematics and Computer Science, Quanzhou Normal University, Quanzhou, Fujian 362000 China; 2 Fujian Provincial Key Laboratory of Data-Intensive Computing, Quanzhou, Fujian 362000 China; 3 School of Advanced Technology, Xi’an Jiaotong-Liverpool University, Suzhou, Jiangsu 215123 China; 4 School of Culture and Creativity, Beijing Normal-Hong Kong Baptist University, Zhuhai, Guangdong 519087 China

**Keywords:** Cultural heritage, Digital heritage, Virtual heritage, Gesture interaction, User experience, Learning effectiveness, Embodied learning, First Writing Ceremony

## Abstract

Gesture-based interactions in a virtual reality (VR) setting can enhance our experience of traditional practices as part of preserving and communicating heritage. Cultural experiences embodied within VR environments are suggested to be an effective approach for experiencing intangible cultural heritage. Ceremonies, rituals, and related ancestral enactments are important for preserving cultural heritage. Kāi Bǐ Lǐ, also known as the *First Writing Ceremony,* is traditionally held for Chinese children before their first year of elementary school. However, gesture-based immersive VR for learning this tradition is new, and have not been developed within the community. This study focused on how users experienced learning cultural practices using gesture-based interactive VR across different age groups and hardware platforms. We first conducted an experiment with 60 participants (30 young adults and 30 children) using the *First Writing Ceremony* as a case study in which gestural interactions were elicited, designed, implemented, and evaluated. The study showed significant differences in play time and presence between the head-mounted display VR and desktop VR. In addition, children were less likely to experience fatigue than young adults. Following this, we conducted another study after eight months to investigate the VR systems’ long-term learning effectiveness. This showed that children outperformed young adults in demonstrating greater knowledge retention. Our results and findings contribute to the design of gesture-based VR for different age groups across different platforms for experiencing, learning, and practicing cultural activities.

## Introduction

Kāi Bǐ Lǐ, or the *First Writing Ceremony*, is a traditional Chinese ritual that marks a child’s first formal writing experience. This ceremony is typically held when a child reaches approximately six or seven years of age, symbolizing a significant milestone in a child’s educational journey, and is often accompanied by family gatherings and festivities. At the start of each school session in ancient China, parents took their children to the *First Writing Ceremony* [1]. Today, some ancient-style *First Writing Ceremonies* are held at heritage sites and elementary schools in China. Parents take their children to attend the ceremony before they are admitted to school. The whole ritual includes several traditional activities, usually donning specific clothing and headgear, bowing to Confucius, receiving a vermilion dot for enlightenment, drumming for wisdom, and writing the Chinese character “Ren (人).” However, these practices vary across provinces in China. For diverse reasons, only a few elementary schools participate in the *First Writing Ceremony*, especially in suburban and rural areas, with some being unaware of the traditional practices.

Gestures are body movements that convey information, and is part of how a culture transmit memories, where such practices are passed on to the next generation. The meaning of a gesture can vary and is often specific to certain situations and backgrounds. Broadly speaking, gestures can be classified into either communication or manipulation. Communicative gestures comprise a series of symbolic movements with a fixed contextual meaning fulfilling a specific informative function [2]. Manipulation gestures comprise hand or arm movements that control the state of objects by moving, pointing, selecting, dragging, dropping, grasping, rotating, scaling, or clicking objects [2]. These categorization does make it easier to structure digitization works in intangible cultural heritage. Based on the different display modes, scenes with mid-air gesture interactions can be implemented for 3D virtual scenes through a traditional desktop display, or virtual reality head-mounted displays (VR HMDs). The recent development of real-time hand tracking for commercial depth-sensing devices (e.g., Leap Motion and Kinect) and VR headsets (e.g., Meta Quest 2 and Pico 4) has allowed various techniques that support hand tracking and a recognition technology in immersive VR, which facilitate hand-gesture interaction with virtual scenes or virtual objects in immersive virtual environments. Recent studies have used hand-tracking interactions to foster realistic connections between the real and virtual hands, allowing for intuitive and natural user experiences [3–9]. These systems immerse users in a virtual environment using HMDs with gesture controls, where they grasp or touch virtual objects with an avatar embodying their actions, which enables cultural heritage communication and its preservation by first-person hands-on practice.

Moreover, gesture-based immersive VR provides new opportunities for embodied learning, which comprise physical engagement [9–12]. These studies represent various explorations of embodied learning, an educational approach that integrates physical movements and sensory experiences to enhance cognitive processes. For example, the use of gestures in mixed reality has been found to help learners practice the Guqin [9]. Gelsomini et al. [10] presented IMAGINE, an immersive smart space that uses full-body movements to improve learning outcomes in primary schools. Tan et al. [11] explored Web AR for cultural heritage education and showed that embodied interactions boosted understanding. Pillat et al. [5] presented a mixed-reality system for STEM education using whole-body interaction that enhanced learning through spatial audio and multisensory input. Overall, these studies contribute to the growing body of research advocating the integration of physicality and interactivity in educational technologies. They demonstrated the potential of embodied learning to offer more intuitive, immersive, and effective learning experiences, particularly in the context of emerging technologies such as immersive VR. For traditional cultural activities, the use of gestures can help simulate digital experiences by mapping them to specific user actions. For example, a traditional ceremony could involve various communicative gestures, such as bowing to Confucius. Furthermore, the grasping gestures can be used to hold objects, such as pens and drumsticks. Both desktop and VR platforms require hand-gesture tracking and recognition technologies.

Despite recent advances in this domain, the adoption of gesture-based input methods in virtual environments remains in its infancy, with challenges regarding real-time feedback, accuracy, and adaptability. These aspects are worth exploring across different user groups. Recent researches [3, 6–8, 13] have shed light on this topic, highlighting the nuances of gestural input on desktop and HMD VR. Desktop VR, often tracks gestures using devices like the Leap Motion, allows a somewhat natural interaction with virtual objects [6, 7, 14]. Recent studies [6, 15] implied that gestures between children and adults can vary significantly in complexity. A study focusing on children’s gestural input in VR hand tracking [15] showed that children’s hand kinematics and goal-directed actions may differ from those of adults. Similarly for HMD VR, their differences in physical capabilities and cognitive development stages must be considered [15–20]. Adults generally have more refined motor skills and spatial awareness, which could influence their ability to perform complex gestures in the HMD VR settings [6, 7, 21]. They may be more adaptable to learning and executing the intricate hand movements required for advanced VR interactions [7]. Conversely, children who are still developing these skills are often more adaptable and may be more willing to engage in the learning process required to master gesture-based interactions [15, 21]. In the following subsections, we present a review of the recent literature on our research topic.

### User experience in VR across different devices

VR is transforming how we engage with digital spaces, for which HMD and Desktop VR create different user experiences [22, 23]. Dede [24] highlighted immersive interfaces, such as VR, can enhance education by offering multiple perspectives, facilitating situated learning, and aiding knowledge transfer. Oberdörfer et al. [25] have studied gamified learning using desktop VR and HMD and evaluated the usability of systems, showing that affine transformations through VR increased flow, preference, and learning in immersive environments. HMD VR has the advantage of making users feel truly present, which can boost user confidence and learning [26].

Feng [27] compared the desktop and HMD VR regarding pedestrian behavior, showed that the desktop VR performed better in wayfinding tasks, but route choices and experiences were comparable. Sousa Santos et al. [28] compared HMD and desktop 3D navigation, finding that users were generally satisfied with VR but performed better on desktops for navigation tasks. Xue et al. [29] evaluated VR against traditional teaching methods in Biology Lab education and found that VR enhanced long-term retention and immersion, with no difference in short-term retention. In addition, Zhao et al. [30] compared desktop and immersive VR virtual field trips to actual field trips for novice geoscience students and found that against desktops, VR increased motivation and presence, but not learning outcomes.

Ai-Lim Lee et al. [16] investigated how desktop and immersive VR impact learning outcomes. The former was found to enhance learning through psychological factors, such as presence and motivation, with the effectiveness moderated by student characteristics. Their study offers a theoretical model for refining VR-based educational strategies. By contrast, immersive VR, while increasing emotional arousal, appears to diminish cognitive engagement, resulting in poorer performance on transfer tests than traditional video lessons [17]. This indicates that an immersive environment, despite its high engagement levels, might overwhelm learners, thereby impeding the cognitive processing essential for effective learning.

### User experience in gesture-based VR across different age groups

Previous research has shown that adults and children interact differently with gesture-based VR systems because their motor skills and cognitive development differ [6–8, 15]. Adults typically find gesture-based interfaces more intuitive and natural, allowing seamless interactions with virtual objects and environments [14]. However, children’s still-developing motor skills and cognitive frameworks may result in a steeper learning curve for certain VR interactions [6, 12, 15]. Furthermore, children’s gestural inputs into VR can be classified into goal-directed actions and hand kinematics, which are crucial for understanding their spatial thinking strategies [15].

Aside from the differences in skill development, previous work has also indicated the effect of age on control and negative feelings. Gesture-recognition accuracy can be challenging for adults, causing frustration and decreased satisfaction [2–4, 6, 28]. The prolonged use of gesture-based VR may also result in discomfort or fatigue owing to the physical demands of making gestures in the air [6, 7]. Research has shown that the immersive quality of VR can lead to increased presence and enjoyment for children, as their imaginations and lower thresholds for the suspension of disbelief can make it easier for them to become engrossed within virtual environments [10, 15]. This immersive aspect can be particularly beneficial for educational content, where VR can provide a rich and interactive learning experience that is both enjoyable and informative [2, 3, 9–12].

Designing gesture-based VR experiences requires a nuanced understanding of the distinct capabilities and needs of adults and children. While adults may value the efficiency and naturalness of gesture interactions [6, 7, 14], children’s experiences may be more dependent on the educational content, the encouragement of imaginative play, and support for their developing physical and cognitive skills [15]. Therefore, to enhance the overall user experience, gesture-based VR systems should consider the different age groups’ unique developmental stages and needs.

### Learning effectiveness of a VR game among different age groups

Previous studies have demonstrated the suitability [31] and short-term learning outcomes [32–34] of VR in facilitating learning activities. Checa and Bustillo [31] demonstrated that VR is suitable for teaching historical knowledge and urban layout. Kilcioglu et al. [32] studied the short- and long-term effects of VR on motor skill learning in children with cerebral palsy. Their results showed a significant short-term effect when including VR in conventional upper-limb function therapies, but the medium- and long-term effects were found to be insignificant. In addition, Martin-Moratinos et al. [35] studied how an educational VR game affected emotion regulation in children with attention-deficit/hyperactivity disorder. They found significant improvements in material organization, working memory, and inhibition, particularly among those who were more engaged in the game. Similar results were observed in older adults, where improvements were observed in motor learning during immersive VR [24]. Overall, these studies illustrate the strength of serious VR games in learning and in promoting motor skill learning in children and older adults.

### Research motivation and contribution

The review of previous studies revealed gaps in this research area, questioning the suitability of gesture-based VR systems (HMD VR and Desktop VR) for learning intangible cultural heritage, and highlighting the potential differences between different age groups. Motivated by the research gaps, we investigated the user experience and the learning effectiveness of a VR system for cultural practices. Specifically, this study aimed to develop an immersive gesture-based VR application for cultural activity learning by embodying players’ hands and bodies to learn traditional actions related to cultural ceremonies from a first-person perspective. As a virtual replication of the traditional practice, we designed and implemented gesture-based VR applications for the *First Writing Ceremony*, adopting both the HMD VR and Desktop VR. For the HMD VR, we implemented gesture-tracking techniques. For the Desktop VR, we used a Leap Motion controller for gesture tracking. Both VR platforms guided users through five scenes of traditional activities relating to the *First Writing Ceremony*: (1) adjusting clothing and headgear, (2) bowing to Confucius, (3) applying the vermilion dot for enlightenment, (4) drumming for wisdom, and (5) writing the Chinese character “Ren (人).” In these virtual environments, body and hand gestures interact with a virtual student avatar, virtual drum, and virtual brush pen to complete the rituals. We empirically investigated and evaluated the differences between young adults and children regarding user experience (play time, usability, presence, control, fatigue, workload, and acceptance) and between two VR platforms (HMD VR and Desktop VR). Additionally, we conducted a follow-up study to understand long-term learning effectiveness in the adult and child groups. The results demonstrated that the participants spent a significantly longer time and perceived a stronger presence in the HMD VR than the Desktop VR. In addition, children felt less fatigued than young adults. Concerning long-term learning effectiveness, the children outperformed the adults exhibiting greater knowledge retention. The results provide insights into the design of gesture-based VR across different VR platforms and age groups for learning cultural activities.

Our study makes three main contributions to the literature. First, we designed and implemented two VR applications for learning the *First Writing Ceremony* from a first-person perspective, using the HMD VR and Desktop VR. Second, we demonstrated the feasibility of mitigating the use of VR controllers in cultural heritage learning, adopting a natural gesture-based interaction method by embodying players’ hands and bodies to learn the gestures of cultural activities. Third, we conducted a comprehensive comparative study to evaluate the difference in user experience between young adults and children when utilizing an HMD *vs* a desktop display, both with gestures as input. Our results revealed significant differences in play time and presence between the two device types as well as disparities in fatigue between young adults and children. Additionally, children demonstrated significantly greater long-term knowledge retention than young adults, after learning with VR systems. These results provide insight into the design of gesture-based VR systems for different user groups.

## Methods

### System design goals

The structural design and implementation of the *First Writing Ceremony* were driven by the following design goals (DGs):

- DG1: To provide an immersive experience of the *First Writing Ceremony* from a first-person perspective.
- DG2: To support embodied gesture interactions using the players’ hands and anatomy to learn the traditional acts related to the *First Writing Ceremony*, including adjusting clothing and headgear, bowing to Confucius, receiving the vermilion dot for enlightenment, drumming for wisdom, and writing the Chinese character “Ren (人).”
- DG3: Adopting a learning-by-doing approach to accommodate various user groups, including children and young adults, and familiarizing them with traditional Chinese customs and rituals associated with the *First Writing Ceremony*.

### Apparatus

The setup comprised Unity (version 2021.3.34. f1c1) and a laptop with an Intel(R) Core (TM) i5-1135G7 @ 2.40 GHz CPU and an NVIDIA GeForce RTX 3050 graphics card for system development. We used MRTK to develop hand-tracking in Unity. For system deployment, we used Pico 4 VR HMD, an all-in-one HMD with a Snapdragon® XR2 processor. It has a binocular resolution of 4320 × 2160 pixels and a field of view of 105°. The headset provides hand-gesture tracking using four cameras with 21 anchor points on one hand. It recognizes the distance of 20–48 cm between the hand and HMD, a field of view of 114 × 132°, and recognition accuracy of approximately 15 mm. For the desktop setup, we used the Leap Motion controller, an optical hand-tracking module designed to capture the intricate movements of the hands and fingers. It enables natural interactions with digital content. It has two cameras for gesture recognition and tracks 27 different hand elements, including bones and joints. It has a tracking range of 25–600 mm, a field of view of 140 × 120°, and a detection accuracy of approximately 0.01 mm at the fingertip position.

### System implementation of the ***First Writing Ceremony***

**Fig. 1:**
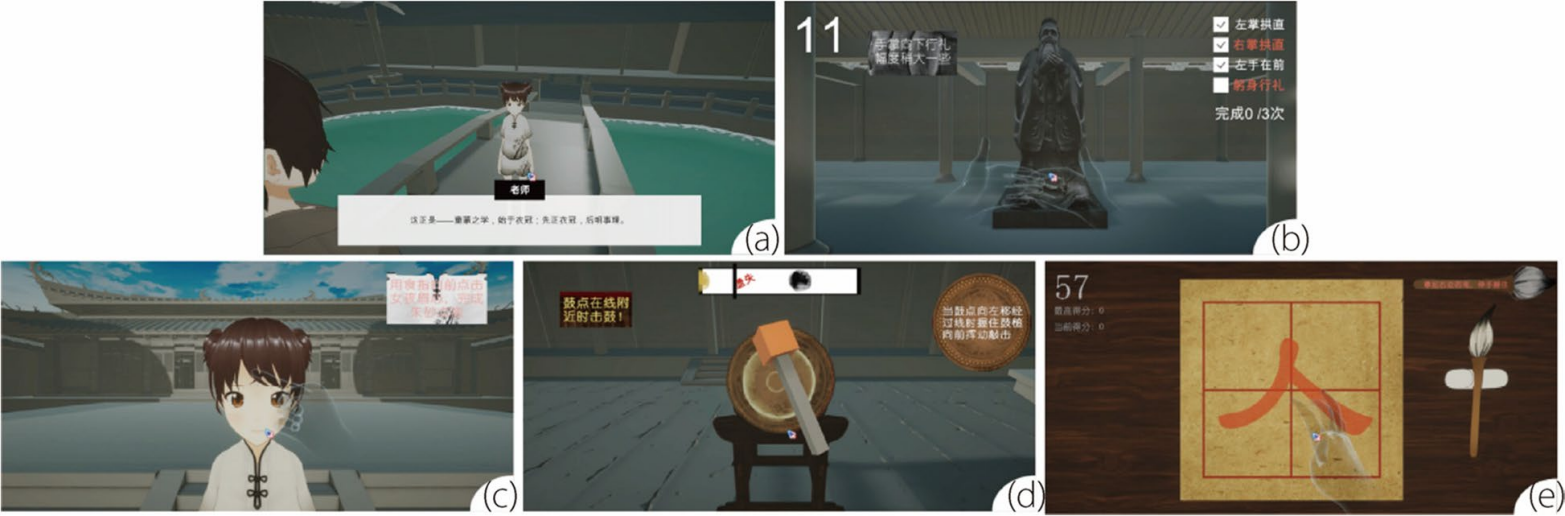
*First Writing Ceremony:* Learning in Desktop VR mode. **a** Adjusting clothing and headgear; **b** Bowing to Confucius; **c** Vermilion dot for enlightenment; **d** Drumming for wisdom; and **e** Writing the Chinese character “Ren (人)”

**Fig. 2:**
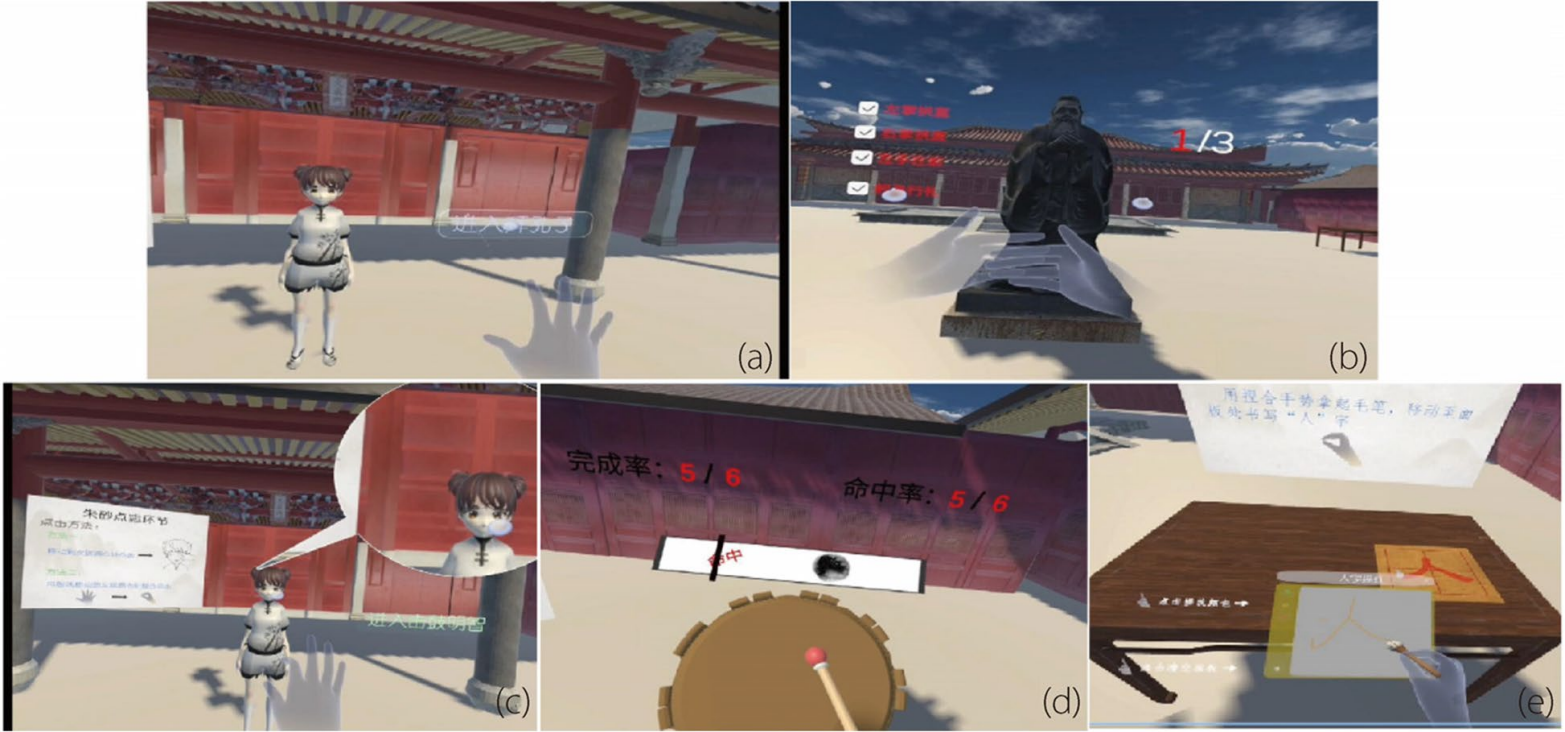
*First Writing Ceremony:* learning in HMD VR mode. **a** Adjusting clothing and headgear; **b** Bowing to Confucius; **c** Vermilion dot for enlightenment; **d** Drumming for wisdom; and **e** Writing the Chinese character “Ren (人)”

Following the practice and reports on the *First Writing Ceremony*, both the Desktop VR (Fig. 1) and HMD VR (Fig. 2) prototype scenarios were divided into five scenes. In designing the two prototypes, we aimed to achieve a balance between recognition accuracy and the natural gestures used in the *First Writing Ceremony*. The gestures were the same for both VR systems. Table 1 lists the gestures used in each of the five scenes. The following sections describe the technical details of the implementation of the virtual ceremony and corresponding gesture controls.

**Table 1:**
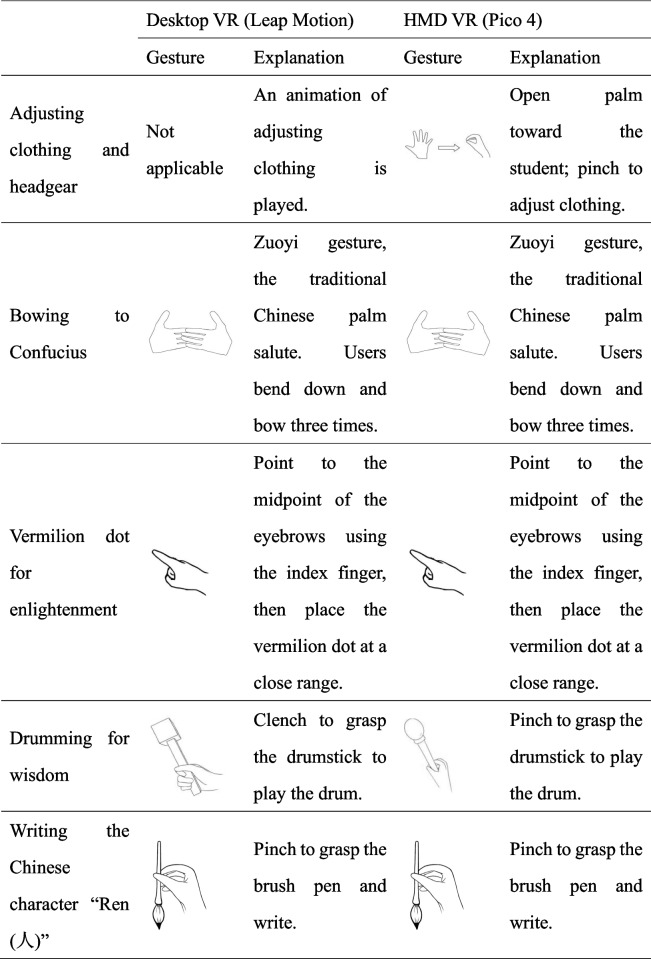
Detailed explanations about the gesture control in the desktop VR and the HMD VR

#### Adjusting clothing and headgear

##### Scene description

Clothing and headgear signify modesty, and more importantly, reflect a person’s spirit and demeanor. This virtual scene simulates the avatar wearing a Chinese school uniform for the first time. After listening to an explanation of wearing traditional Chinese costumes, the user adjusts the avatar’s clothing and learns how to wear the traditional Chinese suit Figs. 1(a) and 2(a) illustrate the two scenes.

##### Gesture explanation

In HMD VR, users open their palms and pinch to adjust the clothing. The Desktop VR has no gestures but plays an animation of adjusting the clothing.

#### Bowing to confucius

##### Scene description

In this scene, the user plays the role of a student. Through auxiliary pictorial text, voice, and gesture prompts, users learn the gesture and required number of bows to Confucius. When the user enters the scene, an introduction to the salutation is illustrated using images and text. A virtual statue of Confucius is placed in the hall. A series of audio instructions are given to the users after each movement, starting from extending the left arm, keeping the thumb straight, and the other fingers together in front of the chest. Next, we extend the right arm, keeping the thumb straight while bringing the other fingers together, and position half of the right hand on top of the left hand. Finally, facing the statue of Confucius, the student bends and bows thrice. Figures 1(b) and 2(b) illustrate these two scenes.

##### Gesture explanation

Users execute the *Zuoyi* gesture by placing one hand over the other. The palms are turned inward with the fingers slightly curled, creating a gentle and respectful appearance. The user then bows three times. The application recognizes gestures and provides real-time visual and audio feedback.

#### Vermilion dot for enlightenment

##### Scene description

This scene includes a student avatar that simulates a newly enrolled pupil. This traditional custom involves placing a vermilion dot on the forehead. This action is explained in text, images, and audio. Figures 1(c) and 2(c) illustrate these two scenes.

##### Gesture explanation

In both Desktop VR and the HMD VR, one index finger approaches the midpoint between the avatar’s eyebrows and then applies the dot by pinching the finger and thumb.

#### Drumming for wisdom

##### Scene description

In this scenario, a virtual hand grasps a drumstick and strikes with it. Collision detection is triggered when the drumstick hits the drum surface. When the drum is struck, it ‘resonates,’ producing a sound the participant can hear. A progress bar displaying the drumbeats is located at the top of the interface; if users hit the drum in time with the beats, the word ‘hit’ will appear, while ‘miss’ will show if they do not. Figures 1(d) and 2(d) illustrate the two scenes.

##### Gesture explanation

To grasp the drumstick in the HMD VR setting, users perform a pinch gesture using the thumb and index finger of one hand. This is similar to the grasping gesture used in the Desktop VR, where users clench their entire hand. The drumming sound is triggered by the drumstick striking the drum surface.

#### Writing the Chinese character “Ren (人)”

##### Scene description

Pupils in the early primary school grades learn Chinese calligraphy. In this scene, the virtual hand grasps the brush pen and writes the Chinese character “Ren (人)” on the writing board. Figures 1(e) and 2(e) illustrate these two scenes.

##### Gesture explanation

Similar to grasping the drumstick, users perform a pinch gesture to grasp the brush pen in both Desktop VR and HMD VR.

### Research questions and hypotheses

Our research aimed to compare and evaluate the user experiences of young adults and children learning a cultural activity using different VR display types. In addition, we explored the long-term learning effectiveness of serious VR games for young adults and children by proposing the following research questions:

1. Does the user experience (including play time, usability, presence, workload, fatigue, and acceptance) with the *First Writing Ceremony* VR systems differ between young adults and children?
2. Does the user experience (including play time, usability, presence, workload, fatigue, and acceptance) with the *First Writing Ceremony* VR systems differ between the HMD VR and Desktop VR?
3. Does the long-term learning effectiveness differ between young adults and children after using the *First-Writing Ceremony* VR systems?

Based on the related work, we proposed the following hypotheses:

1. The user experience with the *First Writing Ceremony* VR system differs between young adults and children.
2. Users will perceive a better user experience (in terms of play time, usability, presence, workload, fatigue, and acceptance) with the *First Writing Ceremony* VR system when using the HMD VR compared to Desktop VR.
3. Long-term learning effectiveness differs between young adults and children after using *First Writing Ceremony* VR systems.

### Experimental procedure

**Fig. 3:**
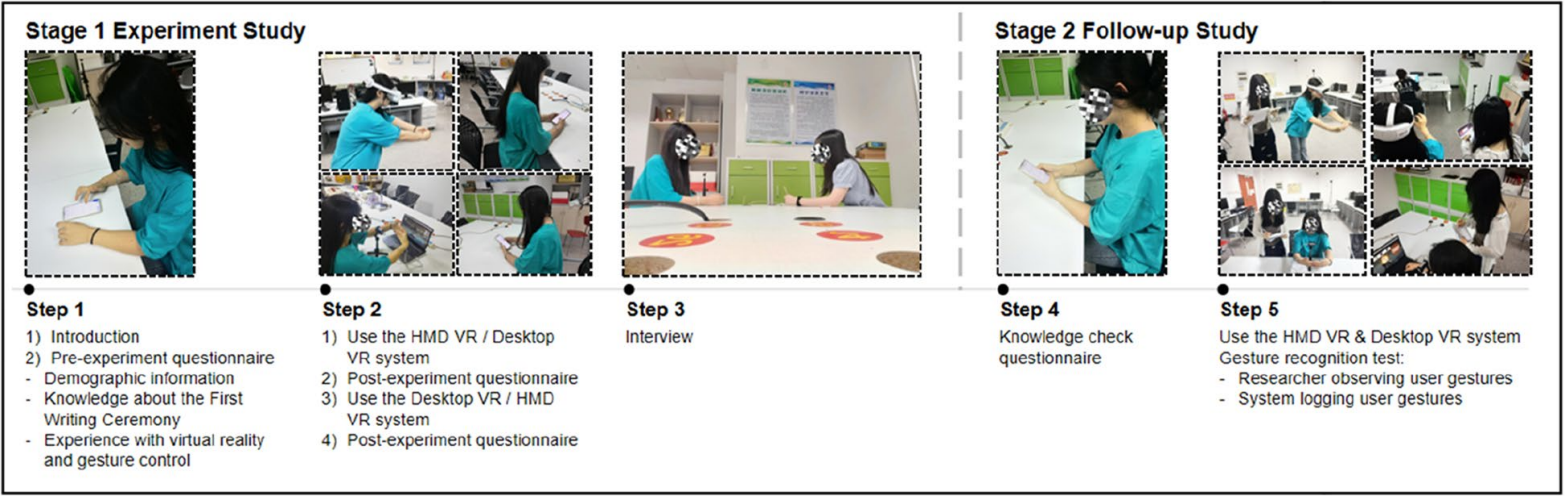
The evaluation comprised two stages: an experiment study and a follow-up study

The evaluation comprised two stages, as illustrated in Fig. 3. First, we conducted an experiment focusing on the differences in the HMD VR and Desktop VR user experience between young adults and children. After eight months (Stage 2), a follow-up study was conducted, including (1) a knowledge check to understand the long-term learning effectiveness between young adults and children and (2) a gesture recognition test to investigate the accuracy between the two age groups and the two device types.

**Fig. 4:**
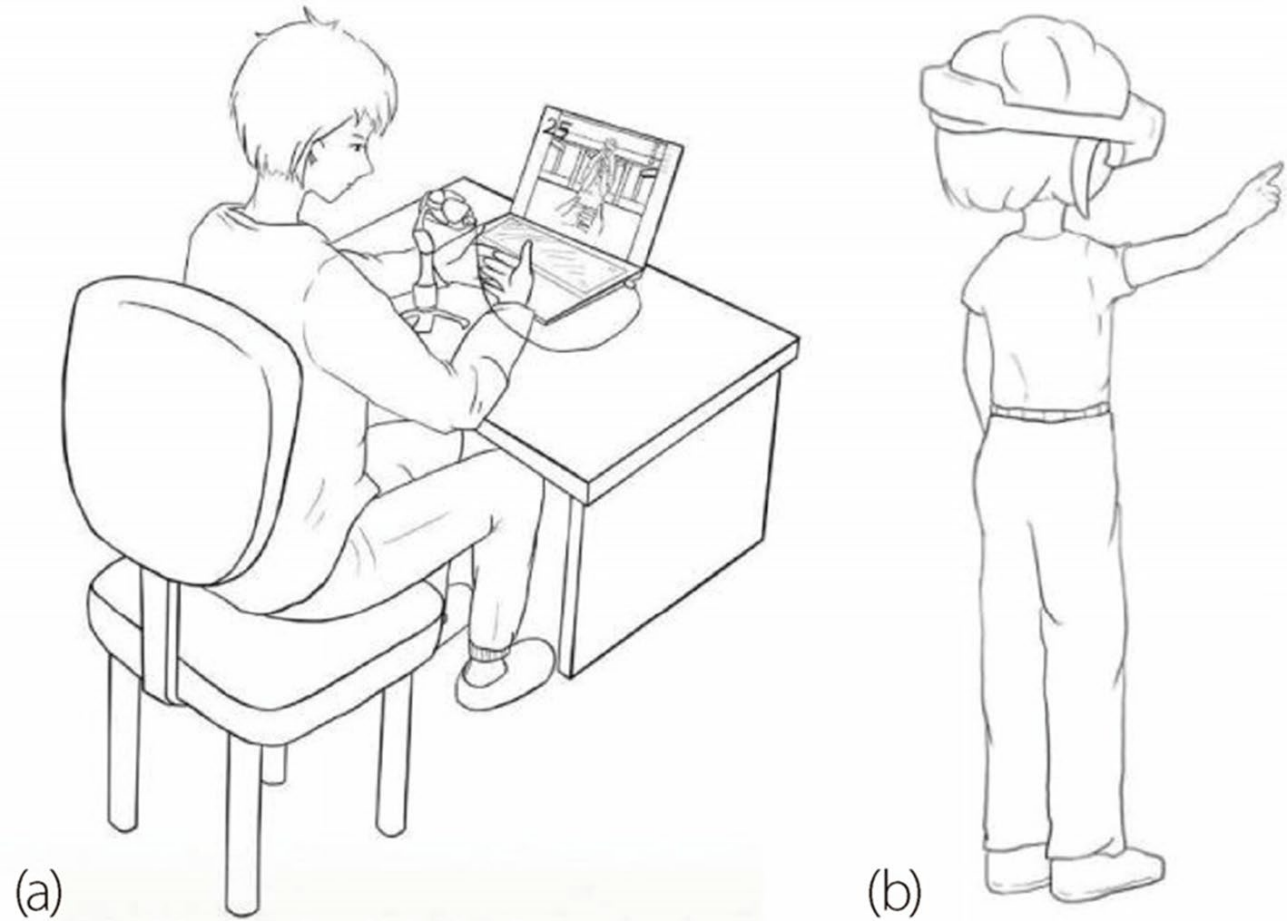
Experimental set up of the two conditions: **a** Desktop VR: a participant sitting in front of a laptop and performing gesture interactions with the Leap Motion; **b** HMD VR: a participant standing and wearing the HMD, performing mid-air gesture interactions

To evaluate the HMD VR and Desktop VR user experience as perceived by children and young adults, we executed a mixed study design, with age group as the between-group variable and device type as the within-group variable. The Desktop VR modality was tested in a non-immersive environment using a laptop equipped with Leap Motion, whereas the HMD VR condition was tested in an immersive environment using a Pico HMD with mid-air hand gesture inputs. Figure 4 shows the two task settings.

For the experimental study, participants first read and signed the experimental consent form, then completed the pre-experiment questionnaire collecting basic demographic information, knowledge of the *First Writing Ceremony*, and their prior experience of VR and gesture control. The experiment received ethics approval from the university’s ethics committee and was conducted under two conditions: HMD VR and Desktop VR. The sequence of the two conditions was counterbalanced [36]; half of the participants used the HMD VR first, whereas the other half began with Desktop VR. After completing the experimental condition, the participants answered a related post-experiment questionnaire and then proceeded to the other condition. After completing both experimental conditions, which lasted for twenty-five minutes per participant, the subjects were interviewed. The participants could withdraw from the study at any time.

After eight months, a follow-up study was conducted to evaluate the long-term learning effectiveness of this educational VR game for children and young adults. The participants were invited to complete a knowledge check to assess their long-term knowledge retention and evaluate their perceived knowledge. In addition, informed by the results and findings from the first stage, the follow-up study also delved into the technical aspects of the gesture recognition accuracy of the two systems. We conducted a gesture-recognition test to determine whether there were any differences between the Pico and Leap Motion systems. The participants were invited to use the same HMD VR and Desktop VR systems while we observed and recorded the times of the intended gesture control and logged the gestures that were successfully recognized in the system. We also invited participants to provide a subjective evaluation of their perceived accuracy using HMD VR and Desktop VR.

### Measurements

The pre-experiment questionnaire comprised demographic questions including gender, age, knowledge of the *First Writing Ceremony*, experience of VR, and experience of natural gesture interaction. When the participants used the HMD VR or the Desktop VR system, the time spent in each session was recorded in the system logs. The post-experiment questionnaire invited the participants to evaluate their experiences over six dimensions: usability, presence, control, workload, fatigue, and acceptance. Metrics for the explicit dimensions of user experience were sourced from validated questionnaires comprising 30 items (see Appendix 1 for details). The answers were given using a 7-point Likert scale.

In the follow-up study, we conducted a knowledge check comprising 12 true/false questions that tested the participants’ knowledge retention in the *First Writing Ceremony*. The questions were randomly sorted. In addition to the objective knowledge questions, we asked six subjective evaluation questions to understand the perceived knowledge of participants, each response being rated on a five-point Likert scale (5 = strongly agree). We also included a gesture recognition test to obtain an in-depth understanding of the experimental user experience. Appendix 2 provides the details of these questions.

#### Play time

Participants’ play time was calculated as the end time minutes the start time in seconds. This shows the total time each participant spent interacting with the five scenes.

#### Usability (U1-U4)

The International Organization for Standardization (ISO 9241–210:2010) defines usability as the extent to which a product can be effectively applied by users to achieve clear goals efficiently and satisfactorily in a specific context. Notably, the System Usability Scale (SUS) [37] is widely used to measure the perceived usefulness, efficiency, effectiveness, and usage of a system. We adopted four items from the SUS to help us assess how easily users understood and learned the ceremony using gestures in a VR setting.

#### Presence (P1-P4)

Presence is defined as the subjective experience of being in one place or environment, even when one is physically situated in another [38, 39]. To assess the extent of immersion the participants perceived in the virtual ceremony, four questions were derived from a Presence Questionnaire [38].

#### Control (C1-C5)

In virtual environments, control indicates the ability to manipulate digital objects accurately or perform actions using input devices such as hand gestures. This is crucial for the user experience since it affects immersion and satisfaction. A good sense of control means that users can execute their intended actions with ease and precision, influenced by the tracking accuracy, environment responsiveness, and an intuitive interface. Good control allows users to play virtual drums or use a virtual brush pen with motions that naturally match their intentions. It also enables users to execute detailed tasks with precision, e.g., dotting the virtual student’s brow. Control in VR involves the user’s actions aligning seamlessly with the system’s responses, creating a satisfactory interaction.

#### Workload (W1-W5)

Workload, often referred to as the cognitive workload, is the amount of mental effort required to perform a task or activity. It can be influenced by various factors such as task complexity, time pressure, and skill level. The NASA Task Load Index (NASA-TLX) [40] has been widely used as a subjective measure to assess human mental workload. In this study, five items from the NASA-TLX were used to measure mental demand, physical demand, temporal demand, performance, and effort, which revealed how much effort they invested in the task.

#### Fatigue (F1-F3)

Visual fatigue is a complex problem in VR owing to its resolution and optical efficiency. Zhang and Jiang [41] compared the differences in visual fatigue between the HMD VR and an iPad, indicating that the VR HMD would cause more eye strain and headaches while the iPad would affect eye dryness more. Three questions were derived from the Chalder Fatigue Scale [42].

#### Acceptance (A1-A9)

To assess how well the participants accepted learning and communicating about the ceremonies and cultural activities in gesture-based virtual environments, we derived nine items on acceptance from refs. [43] and [44], which included positive reception and belief in the virtual environment. Acceptance is crucial for immersive learning, such as mastering ceremonial rituals, in which users must find virtual interactions credible and worthwhile. When users accept a virtual setting, they are more likely to be fully engaged, leading to a deeper understanding and appreciation of cultural content. For example, virtual participation in rituals, such as bowing to Confucius, can be seen as an effective and enriching way to learn about and experience cultural ceremonies.

#### Long-term learning effectiveness (K1-K12, PK1-PK6)

To assess the VR serious game’s long-term learning effectiveness, we included 12 true or false objective questions to test long-term knowledge retention. In addition, six subjective questions on perceived knowledge were included to assess perceived understanding about the overall ceremony procedure and the five specific scenes: adjusting the clothing and headgear, bowing to Confucius, placing the vermilion dot for enlightenment, drumming for wisdom, and writing the Chinese character “Ren (人).” The questions covered the knowledge conveyed in the *First Writing Ceremony*, which was visualized in the system, explained using text and audio, or embodied in hand and body gestures.

#### Gesture recognition accuracy (PA1-PA6)

To obtain both objective and subjective measures of gesture recognition accuracy using the VR devices, we specifically observed the participants’ intended gesture interactions (*N*) and recorded the system logs of gesture recognition (*L*). The gesture-recognition accuracy was calculated using *N/L*, namely, the gestures performed by the participant divided by the gestures detected by the system. In addition, we included six questions regarding the perceived accuracy. These questions were rated on a five-point Likert scale ranging from strongly disagree (1) to strongly agree (5).

### Participants

The experiment involved two groups of participants (60 each). Thirty participants were from a local primary school (16 females and 14 males, aged 10.1 ± 0.8). The adult group comprised 9 females and 21 males aged 18–24 years (20.4 ± 1.3). Among all the participants, 19 adults had no VR experience, 21 had no natural gesture interaction experience, and 7 knew nothing of the *First Writing Ceremony.* In the child group, 22 participants had no VR experience, 21 had no natural gesture experience, and 19 knew nothing of the *First Writing Ceremony*. In the follow-up study, a subset of the experimental group accepted the study invitation (*n* = 32), including 16 children (9 females, 7 males, aged 10.6 ± 0.51) and 16 young adults (5 females, 11 males, aged 21.6 ± 0.89).

## Results

### Experimental study: understanding user experience

We obtained 120 sets of system logs as objective performance measures and subjective questionnaires (2 devices × 60 participants) for the experiment. The data was analyzed using IBM SPSS Statistics software. Our study followed a 2 × 2 mixed design, with age group (children and young adults) as the between-group variable and device type (Desktop VR and HMD VR) as the within-group variable. A two-way ANOVA analyzed the main and interaction effects.

#### System logs and post-experiment questionnaires

**Fig. 5:**
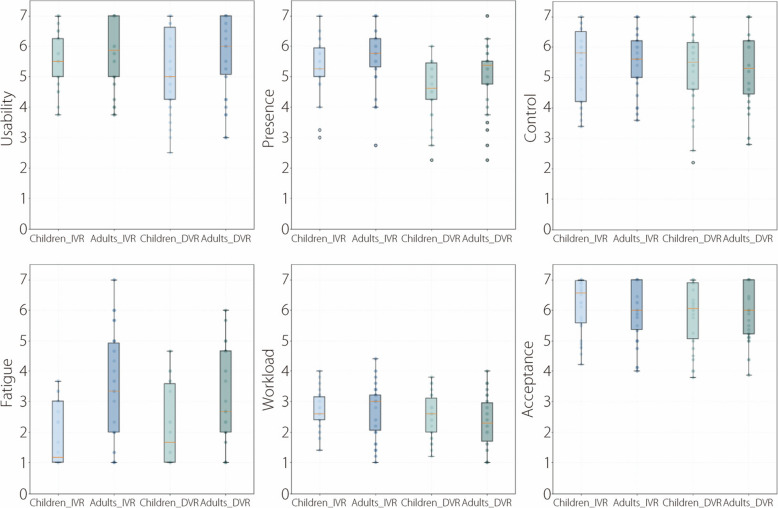
Boxplot comparisons of subjective user experience between children and adults in immersive HMD VR and Desktop VR

The descriptive statistics of the seven dimensions of the user experience are presented in Table 2. The six subjective dimensions of the UX are shown in Fig. 5.

**Table 2:** Descriptive statistics of user experience

Variable	Group	Device	Minimum	Median	Maximum	Mean ± SD
Play time	Adult	HMD VR	108.0	339.5	720.0	**361.2 ± 143.4**
		Desktop VR	127.0	269.5	600.0	**285.9 ± 97.5**
	Child	HMD VR	240.0	350.0	530.0	**365.9 ± 82.8**
		Desktop VR	193.0	237.5	464.0	**254.3 ± 58.6**
Usability	Adult	HMD VR	3.8	5.9	7.0	5.7 ± 1.2
		Desktop VR	3.0	6.0	7.0	5.8 ± 1.3
	Childr	HMD VR	3.8	5.5	7.0	5.6 ± 0.9
		Desktop VR	2.5	5.0	7.0	5.2 ± 1.4
Presence	Adult	HMD VR	2.8	5.8	7.0	**5.7 ± 1.1**
		Desktop VR	2.3	5.4	7.0	**5.0 ± 1.1**
	Child	HMD VR	3.0	5.3	7.0	**5.3 ± 1.0**
		Desktop VR	2.3	4.6	6.0	**4.6 ± 1.0**
Control	Adult	HMD VR	3.6	5.6	7.0	5.5 ± 1.0
		Desktop VR	2.8	5.3	7.0	5.3 ± 1.3
	Child	HMD VR	3.4	5.8	7.0	5.4 ± 1.2
		Desktop VR	2.2	5.5	7.0	5.3 ± 1.3
Fatigue	Adult	HMD VR	1.0	3.3	7.0	**3.4 ± 1.8**
		Desktop VR	1.0	2.7	6.0	**3.0 ± 1.6**
	Child	HMD VR	1.0	1.2	3.7	**1.9 ± 1.1**
		Desktop VR	1.0	1.7	4.7	**2.2 ± 1.3**
Workload	Adult	HMD VR	1.0	3.0	4.4	2.7 ± 0.9
		Desktop VR	1.0	2.3	4.0	2.3 ± 0.9
	Child	HMD VR	1.4	2.6	4.0	2.7 ± 0.6
		Desktop VR	1.2	2.6	3.8	2.6 ± 0.7
Acceptance	Adult	HMD VR	4.0	6.0	7.0	6.0 ± 1.0
		Desktop VR	3.9	6.0	7.0	6.0 ± 0.9
	Child	HMD VR	4.2	6.6	7.0	6.2 ± 0.9
		Desktop VR	3.8	6.1	7.0	5.9 ± 1.1

Dimensions with statistically significant differences are highlighted in bold

##### Play time

The device exerted a significant main effect on play time, F(1, 116) = 25.974, *P* < 0.001, η^2^ = 0.183. Age group exerted no significant main effect on play time, F(1, 116) = 0.535, *P* = 0.466, η^2^ = 0.005; and there was no significant interaction between device and age group, F(1, 116) = 0.976, *P* = 0.325 η^2^ = 0.008. Specifically, participants spent significantly more time using the HMD VR (365.97 ± 116.97) than the Desktop VR (264.59 ± 74.40).

##### Usability

The device exerted no significant main effect on usability, F(1, 116) = 1.003, *P* = 0.319, η^2^ = 0.009; Age group exerted no significant main effect on usability, F(1, 116) = 2.829, *P* = 0.095, η^2^ = 0.024; and there was no significant interaction between device and age group, F(1, 116) = 1.160, *P* = 0.284, η^2^ = 0.010.

##### Presence

The device exerted a significant main effect on presence, F(1, 116) = 14.120, *P* < 0.001, η^2^ = 0.109. Age group exerted no significant main effect on presence, F(1, 116) = 3.658, *P* = 0.058, η^2^ = 0.031; and there was no significant interaction between device and age group, F(1, 116) = 0.041, *P* = 0.840, η^2^ = 0.000. Specifically, the participants perceived a significantly greater presence when using the HMD VR (5.5 ± 1.02) than when using the Desktop VR (4.8 ± 1.03).

##### Control

The device exerted no significant main effect on sense of control, F(1, 116) = 0.830, *P* = 0.364, η^2^ = 0.007; Age group exerted no significant main effect on sense of control, F(1, 116) = 0.015, *P* = 0.904, η^2^ = 0.000; and there was no significant interaction between device and age group, F(1, 116) = 0.045, *P* = 0.832, η^2^ = 0.000.

##### Fatigue

Age group exerted a significant main effect on fatigue, F(1, 116) = 18.886, *P* < 0.001, η^2^ = 0.140. The device exerted no significant main effect on fatigue, F(1, 116) = 0.084, *P* = 0.773, η^2^ = 0.001; and there was no significant interaction between device and age group, F(1, 116) = 1.441, *P* = 0.232, η^2^ = 0.012. Specifically, children reported significantly less fatigue (2.04 ± 1.20) than did adults (3.21 ± 1.69).

##### Workload

The device exerted no significant main effect on workload, F(1, 116) = 3.537, *P* = 0.063, η^2^ = 0.030; Age group exerted no significant main effect on workload, F(1, 116) = 0.738, *P* = 0.392, η^2^ = 0.006; and there was no significant interaction between device and age group, F(1, 116) = 0.820, *P* = 0.367, η^2^ = 0.007.

##### Acceptance

The device exerted no significant main effect on acceptance, F(1, 116) = 0.691, *P* = 0.408, η^2^ = 0.006; Age group exerted no significant main effect on acceptance, F(1, 116) = 0.028, *P* = 0.867, η^2^ = 0.000; and there was no significant interaction between device and age group, F(1, 116) = 0.771, *P* = 0.382, η^2^ = 0.007.

#### Interview findings

To understand more deeply the two groups’ preferences in experimentally utilizing the gesture-based VR systems, our semi-structured interview addressed two questions: (1) Which method is preferred for learning about the *First Writing Ceremony*? and (2) Can you explain why you made this choice?

Of the 60 participants, 58 reported a preference for the HMD VR over the Desktop VR, citing that the immersive experience was more engaging, provided a greater sense of presence, and allowed more natural interactions. Participant P19 said: “*The VR headset allows more natural interactions, making it easier to perform the bowing actions to Confucius*.” P26 also commented that “*The body and gesture operations wearing the headset are easier to control and simple*.” Similarly, P55 (adult) indicated that the HMD VR provided a better experience than the Desktop VR, as it could help achieve higher operational success rates in gesture recognition. P17 commented that the VR HMD provided a greater sense of immersion and more realistic images. However, two adults (P32 and P44) experienced discomfort, noting negative feelings caused by the weight of the HMD, and slight dizziness. These participants preferred the Desktop VR.

Aside from the sense of immersion, participants also commented favorably on the HMD VR regarding body involvement and feeling closer to traditional culture. P19 commented that “*Usually, we can only watch but couldn’t experience this cultural activity. However, with this VR application, we can truly learn it immediately, practice it immediately, and feel it immediately. Previously, I wasn’t interested in this kind of culture, but learning through this VR application I felt being there, and practicing it there. It is similar to reality and fun; makes me more engaged.*” P58 echoed this view, noting that “*Our daily lives are quite distant from calligraphy culture, as we usually rely on electronic devices. However, VR technology has successfully connected calligraphy culture with electronic devices, allowing us to immerse ourselves in the charm of calligraphy. I believe this is an effective measure for preserving this cultural heritage.*” These findings demonstrate the effectiveness of VR applications in the dissemination of cultural practices.

In summary, most participants preferred using the HMD VR because of its realistic images, immersion, and higher success rates in gesture recognition compared with Desktop VR. However, two adults mentioned discomfort and simulator sickness when using the HMD VR. The interview findings indicated that empirically validating the gesture recognition accuracy of the two device types, was essential. The participants further identified that the gesture-based embodied learning process was more fun and engaging, thus effectively sharing traditional cultural activities with young adults and children.

### Follow-up study: understanding learning effectiveness

The follow-up study utilized 64 sets of objective and subjective questionnaires (2 groups × 32 participants). The data analysis employed IBM SPSS Statistics software. The participants’ self-reported learning effectiveness data conformed to a normal distribution (*P* = 0.157), but the knowledge check data deviated (*P* < 0.001). Therefore, we conducted an independent samples *t*-test for the self-reported questionnaire data and a Mann-Whitney U test for the knowledge check data.

#### Long-term learning effectiveness

**Fig. 6:**
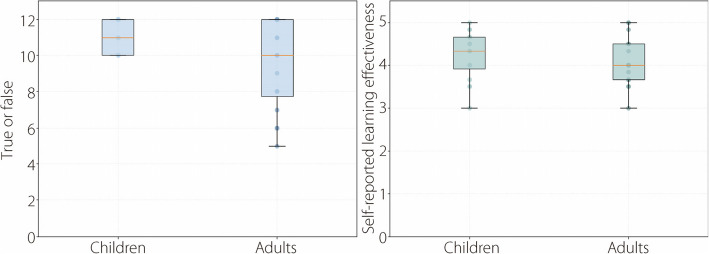
Boxplot comparisons of the knowledge check results and self-reported learning effectiveness between children and adults

Descriptive statistics of the learning effectiveness results are presented in Table 3 and shown in Fig. 6.

**Table 3:** Long-term learning effectiveness between young adults and children

Measurement	Group	Minimum	Median	Maximum	Mean ± SD
Knowledge check (true or false questions)	Adult	5	10	12	9.4 ± 2.5
	Child	10	11	12	11.1 ± 0.9
Self-reported learning effectiveness	Adult	3	4	5	4.1 ± 0.6
	Child	3	4.3	5	4.2 ± 0.6

An independent samples *t*-test showed no significant difference between children and young adults regarding self-reported learning effectiveness, t(30) = -0.720, *P* = 0.477, d = –0.263. However, a significant difference was found between children and young adults in the knowledge check with true-or-false questions (z = -1.673, *P* = 0.094, d = 3.09). Children (11.1 ± 0.9) scored significantly higher than the young adults (9.4 ± 2.5), indicating greater knowledge retention.

#### Gesture recognition accuracy

**Fig. 7:**
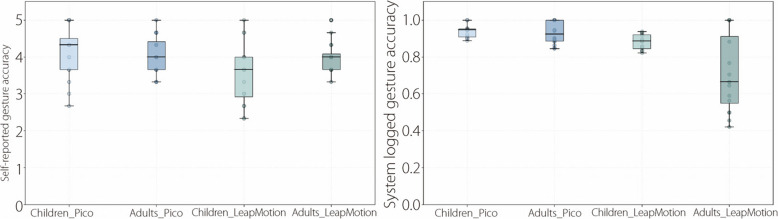
Boxplot comparisons of the self-reported gesture recognition accuracy results and system-logged gesture recognition accuracy results between children and adults in immersive HMD VR and Desktop VR

A two-way ANOVA evaluated the effects of age group and device type on gesture recognition accuracy. The descriptive statistics of the objective and subjective accuracy results are presented in Table 4 and Fig. 7.

**Table 4:** Gesture recognition accuracy between young adults and children regarding HMD VR (Pico) and Desktop VR (Leap Motion)

Variable	Group	Device	Minimum	Median	Maximum	Mean ± SD
Self-reported gesture recognition accuracy	Adult	HMD VR	3.33	4.00	5	4.08 ± 0.49
		Desktop VR	3.33	4.00	5	3.98 ± 0.43
	Child	HMD VR	2.67	4.33	5	4.13 ± 0.72
		Desktop VR	2.33	3.67	5	3.54 ± 0.83
System logged gesture recognition accuracy	Adult	HMD VR	0.85	0.92	1	0.93 ± 0.06
		Desktop VR	0.42	0.67	1	0.71 ± 0.21
	Child	HMD VR	0.89	0.95	1	0.94 ± 0.04
		Desktop VR	0.82	0.89	0.94	0.88 ± 0.04

##### Self-reported gesture recognition accuracy

The results showed that the device exerted a significant main effect on self-reported gesture recognition accuracy, F(1, 60) = 4.584, *P* = 0.036, η^2^ = 0.071. Age group exerted no significant main effect on self-reported gesture recognition accuracy, F(1, 60) = 1.536,* P* = 0.220, η^2^ = 0.025; and no significant interaction between device and age group, F(1, 60) = 2.229, *P* = 0.141, η^2^ = 0.036. Specifically, the participants reported significantly higher gesture recognition accuracy using HMD VR (4.10 ± 0.61) than Desktop VR (3.76 ± 0.69).

##### System-logged gesture recognition accuracy

The results showed that the device exerted a significant main effect on system-logged gesture recognition accuracy, F(1, 60) = 24.989, *P* < 0.001, η^2^ = 0.294. Age group also had a significant main effect on system-logged gesture recognition accuracy, F(1, 60) = 10.568, *P* = 0.002, η^2^ = 0.150. The interaction between device and age group was also significant, F(1, 60) = 8.157, *P* = 0.006, η^2^ = 0.120. Specifically, we found that gesture interactions using HMD VR showed significantly greater recognition accuracy (0.94 ± 0.04) than those using Desktop VR (0.80 ± 0.17). In addition, children’s gesture interactions showed significantly greater recognition accuracy (0.91 ± 0.04) than young adults (0.82 ± 0.14).

## Discussion

This study investigated the user experience and long-term learning effectiveness of embodied gesture-based VR for the *First Writing Ceremony*. Specifically, we explored the perceived user experience across different age groups using HMD and Desktop VR as well as the comparative long-term learning effectiveness for young adults and children. In this section, we discuss the hypothesis-testing results and design implications.

### Overall user experience

In response to RQ1 regarding the young adults’ and children’s user experiences, no significant differences were found in play time, usability, presence, control, workload, or acceptance. However, a difference in perceived fatigue between the two age groups was noted; the children were less prone to fatigue than the young adults. The interview results indicated that young adults encountered issues in gesture recognition when using Leap Motion in Desktop VR. This finding inspired us to obtain an in-depth understanding of the gesture recognition accuracy in a follow-up study.

Comparing the HMD-VR and Desktop-VR user experience (RQ2), our study showed significant differences in play time: both adults and children spent more time using HMD VR than Desktop VR. In addition, our study showed that HMD VR imbued a significantly greater presence than Desktop VR. These results illustrate that compared to Desktop VR, HMD VR sustained user engagement better and yielded a significantly longer play time. It also provides a great sense of being in a virtual ceremony. No statistically significant differences between the HMD VR and Desktop VR were found in usability, control, workload, fatigue, or acceptance.

Investigating the long-term learning effectiveness for adults and children (RQ3) revealed intriguing insights. While both groups reported similar levels of self-perceived learning effectiveness, a more in-depth analysis of their performance in knowledge assessment revealed a notable difference. The following subsection discusses the implications of our findings.

### Reflections on the evaluation results

#### The adults’ and children’s user experiences (H1)

Based on the research findings, we partially accept hypothesis H1, which posits differences in the user experience between young adults and children. Specifically, the significant differences were in fatigue, indicating that children may be less prone to VR fatigue than young adults. This finding is crucial because fatigue can negatively impact user experience and learning effectiveness. Being typically more energetic, children may exhibit different fatigue patterns compared to adults, who may tire more quickly owing to various factors such as physical fitness, attention span, and the cognitive load associated with VR interactions. However, other aspects, such as play time, usability, presence, control, workload, and acceptance, showed no significant differences between the two age groups. This suggests that while fatigue levels may vary, overall engagement, ease of use, sense of being in the virtual environment, ease of operation, mental effort, and acceptance of the VR experience are similar for both children and young adults.

For the HMD VR system, the young adults reported simulator sickness. This may have caused discomfort and negative feelings, leading to higher fatigue in this demographic group. Additionally, children have physical strength advantages [45], which contribute to their lower levels of fatigue compared with adults. Moreover, children tolerate fully immersive 3D VR gameplay with less discomfort than adults [46]. However, the user experience of VR is not solely determined by age, a perspective shared by previous work [47] that emphasizes the role of individual differences in VR acceptance and satisfaction.

Using a Desktop VR system, our follow-up study examined gesture recognition accuracy in detail and found that users, particularly young adults, evidenced significantly lower gesture recognition accuracy when using Leap Motion. This may have caused a disadvantage in the user experience with Desktop VR compared with HMD VR. In addition, unlike the involvement of full-body gestures in HMD VR, the Desktop VR system mainly involves the users’ hand gestures. This may also weaken user engagement in interactions. Nevertheless, the interview results reflect that two participants (P32 and P44) highlighted the discomfort associated with wearing the HMD, an issue indicated in a previous study [48].

#### User experience with HMD VR and desktop VR (H2)

Based on our findings, we partially accept H2, which suggests that users will have a better user experience with HMD VR than with Desktop VR. This claim is supported by the play time and presence findings. This study indicates that young adults experienced longer play times and a higher sense of presence in HMD VR environments. Of the 30 young adults, 28 expressed a preference for HMD VR in the interviews, citing higher successful hand-tracking rates and a greater sense of presence. This finding is consistent with previous work [26], which indicated a higher sense of spatial presence and immersion when using HMD VR than when using Desktop VR. However, there were no significant differences in the usability, control, fatigue, or acceptance between the two types of devices. Our findings suggest that while HMD VR may offer a more immersive experience with a longer play time, it does not necessarily provide advantages regarding ease of use, control, fatigue levels, or user acceptance compared to Desktop VR.

Overall, both age groups spent more time and perceived greater presence using the HMD VR than the Desktop VR. This preference could be due to the immersive and engaging nature of HMD VR, which was supported by the interview responses of participants (e.g., P17, P26, P19, and P55). This finding also aligns with a previous work [17], which highlighted the potential of HMD VR in providing more engaging and enjoyable experiences, particularly for younger users. Children’s endurance [45, 46] in using HMD VR demonstrated more opportunities for ceremony learning. Furthermore, the intuitiveness of gesture control in VR allowed users to participate from a first-person perspective and effectively understand the connotations and operations of cultural practices.

#### Learning effectiveness between adults and children (H3)

During the knowledge check, we found that children significantly outperformed adults. Out of 12 true-or-false questions, every child was able to correctly answer at least 10 questions, demonstrating a strong understanding and knowledge retention resulting from learning with the VR systems. In contrast, some adults struggled, with a few who only managed to answer five questions correctly. This disparity in performance suggests that educational VR games may have greater potential for enhancing learning outcomes in children than in adults. The immersive and interactive nature of VR environments can be particularly effective for younger learners, fostering better engagement and understanding of the content. Moreover, the findings imply that children may experience greater knowledge retention in the long term when exposed to VR educational tools. This could be attributed to their adaptable learning styles and the captivating nature of their VR experiences, which may have enhanced memory consolidation. Overall, these results highlight the importance of age differences when designing educational interventions. By tailoring educational VR games to leverage the strengths of younger audiences, educators and developers can maximize learning effectiveness and knowledge retention in children, potentially leading to improved educational outcomes.

In conclusion, our findings contribute to the growing body of literature on VR by suggesting that age and VR device type may not be the primary determinants of user experience. Instead, our results underscore the importance of considering individual differences, task characteristics, and the specific affordances of VR platforms in understanding and optimizing the user experience in VR environments. In addition, our research demonstrated the feasibility and effectiveness of gesture-based immersive VR for experiencing, learning, and practicing cultural activities.

### Design implications

Based on the study findings, several guidelines can be proposed for designing VR applications for young adults and children.

#### Enhancing presence

Higher presence scores for HMD VR among adults and children indicate that immersive environments are highly valued. Designers should consider balancing immersion and realism without compromising user comfort and ease of use. This would involve fine-tuning the visual and auditory elements to create a more engaging experience without overwhelming users.

#### Fatigue and gesture control

This study highlighted the notable differences in fatigue levels between adults and children. To address this issue, designers should consider optimizing session lengths and incorporating breaks to reduce fatigue and improve user comfort during extended use. Incorporating scheduled breaks into the VR experience enhances user comfort and reduces the likelihood of fatigue. In addition, our follow-up study revealed a difference in gesture recognition accuracy between the two age groups. Designers should consider designing content that capitalizes on children’s strength in gesture recognition and their lower susceptibility to fatigue. This can lead to more effective learning outcomes and improved knowledge retention.

#### Long-term learning effectiveness

Children outperformed young adults regarding long-term learning effectiveness. This finding highlights the significant potential of VR as a tool to enhance cultural knowledge and activity-based learning among younger users. Given these findings, educators and stakeholders should consider strategies for effectively integrating VR into children’s education, such as exploring ways to incorporate VR games into existing curricula and providing training and resources for educators on effective VR-use in the classroom. Educational institutions can also collaborate with VR game developers to create tailored content that meets students’ specific learning objectives.

#### Device-agnostic interaction

Although we strived to adopt similar gesture-based interactions for the two device types, we still found a significant difference in the user experience. Current user experience is largely affected by display features, notably the immersive features of VR. In addition, HMD VR allows body gestures, whereas Desktop VR based on Leap Motion supports only arm-constrained gestures. Designers should consider the unique capabilities and limitations of each platform when creating VR experiences: maximizing immersive qualities by incorporating a variety of body gestures in HMD VR, simplifying tasks to make them more manageable within the constraints of Desktop VR, and integrating additional input methods such as keyboard shortcuts or mouse controls to enhance the overall user experience.

#### Age-neutral design

While we observed some differences between adults and children in their user experience (e.g., fatigue), most aspects of user experience showed no significant differences. Designers must create inclusive applications that cater to a wide age range. This approach can enhance accessibility and enjoyment for all users, thereby avoiding unnecessary segmentation based on age.

### Limitations

Although this study attempted to consider various aspects of the learning process using HMD VR and desktop VR in as much detail as possible, some were beyond the scope of this research. The first limitation concerns the design and evaluation of gesture-based VR for this study topic. This research was based on a commercially popular VR HMD and Leap Motion for hand tracking. In using the built-in hand-tracking modules its precision and latency may have affected the results [26]. In addition, the play time recorded reflects the time spent in each session. In future studies, we plan to increase granularity and record more detailed behavioral data, such as the time spent in each scene, the number of errors, the number of actions, and hand movements. This would allow us to collect more feedback across different platforms and age groups. Furthermore, owing to differences in Pico and Leap Motion’s inbuilt gesture sets, the gesture interactions were somewhat inconsistent and actions such as bowing, pointing, and grasping, varied slightly. In the interview, it was also mentioned that Pico appeared to be more accurate and responsive than Leap Motion. This limitation is inherent to hardware devices. Finally, previous work indicated that the prior experience in VR and gesture interactions of participants may affect their experiences [44]. In the future, we plan to invite more participants with diverse prior experiences to obtain insights into individual and group differences in user experiences of VR platforms.

## Conclusions

To investigate gesture-based embodied learning in VR for cultural activity experiences across different platforms, we designed and implemented two gesture-based applications for *KaiBiLi*, also known as the *First Writing Ceremony*. We developed a Desktop version with a Leap Motion controller and a HMD version using a Pico VR headset with mid-air gesture control. Sixty participants (30 young adults and 30 children) participated in the user experience evaluation. The results showed significant differences in play time and presence between the HMD and desktop VR. In addition, the children felt less fatigued than the young adults. Our follow-up study highlighted the longer-term effectiveness of the VR learning systems among the children than that of the young adults. The study also discusses the disparity in gesture-recognition accuracy between age groups and between the two device types. Our results and findings will contribute to the design of gesture-based VR for different age groups across different platforms for participants to experience, learn, and practice cultural activities.

## Data Availability

The datasets used and analyzed in the current study are available from the corresponding author upon reasonable request.

## Appendix 1

Questions measuring the explicit user experience of the *First Writing Ceremony* using HMD VR and Desktop VR. *Italic items* are reverse coded.

**Table:**

Number	**Question**
*U1*	*I found the system unnecessarily complex.*
U2	I thought the system was easy to use.
U3	I would imagine that most people would learn to use this system for *First Writing Ceremony* very quickly.
U4	I felt very confident using the system.
P1	I had a sense of “being there” in the VR scenes.
P2	I felt natural using my hands and body to interact with virtual scenes.
*P3*	*I felt consciously aware of being in the real world whilst playing.*
P4	The virtual hands were felt like my real hands.
C1	I felt I was in good control of the virtual drumstick.
C2	I felt I was in good control of the virtual brush pen.
C3	I felt I was in good control of bowing down to Confucius.
C4	I felt I was in good control of placing a red dot on the brow of the virtual student.
C5	I felt I was in good control of changing the clothes of virtual student.
W1	Mental Demand: How mentally demanding was the task?
W2	Physically Demand: How physically demanding was the task?
W3	Temporal Demand: How hurried or hushed was the pace of the task?
*W4*	*Performance: How successful were you in accomplishing what you were asked to do?*
W5	Effort: How hard did you have to work to accomplishing your level of performance?
F1	I will feel tired if I play too long time.
F2	I suffered from fatigue during my interaction with the virtual environment.
F3	I felt tired with the overall experience in the VR ceremony.
A1	I think that it is an effectiveness approach for learning ceremony of bowing down to Confucius.
A2	I think that it is an effectiveness approach for learning ceremony.
A3	I think that it is an effectiveness approach for communicating ceremony.
A4	I think that interacting directly with the virtual environment using the participant’s hands and body is a very effective way to learn rituals.
A5	I love being involved in culture in this way.
A6	I would like to share my experience with my friends around me.
A7	I would like to experience more cultural activities in this way.
A8	It is interesting to experience *First Writing Ceremony* in Virtual Reality.
A9	It is impressive to experience *First Writing Ceremony* in this type of display in virtual reality.

## Appendix 2

A knowledge check with true or false questions (K1-K12) to measure the long-term learning effectiveness of the *First Writing Ceremony* using HMD VR and Desktop VR. *Italic items* are false statements. PK1-PK6 measure participants’ self-reported learning effectiveness; PA1-PA6 measure participants’ self-reported gesture recognition accuracy using the Pico VR HMD and Leap Motion for Desktop VR.

**Table:**

Number	**Question**
K1	The *First Writing Ceremony* is a traditional ritual for Chinese children when they began their schooling in ancient times.
K2	“Adjusting clothing and headgear” requires students to adjust their clothing, symbolizing a proper attitude toward learning.
*K3*	*“Vermilion dot for enlightenment” is usually placed the dot on the student’s nose.*
K4	Bowing to Confucius involves three bows.
*K5*	*The First Writing Ceremony is typically held when children graduate from kindergarten.*
K6	Writing the Chinese Character “Ren” in the ceremony aims to teach students the principle of learning to be a person.
*K7*	*When bowing to Confucius, hands should be placed at the sides of the body.*
*K8*	*“Drumming for wisdom” is meant to live up the ceremony atmosphere with the drumbeat.*
*K9*	*Writing the Chinese Character “Ren” in the ceremony is intended to practice calligraphy skills.*
*K10*	*“Adjusting clothing and headgear” refers to the teacher helping students put on Hanfu properly.*
K11	In the “drumming for wisdom,” the louder the drumbeat, the greater the student’s aspirations.
K12	The “vermilion dot for enlightenment” is placed on the forehead, symbolizing “opening the third eye” and enlightening the mind.
PK1	I remember the procedure of the *First Writing Ceremony*.
PK2	I know how to bow to Confucius in the *First Writing Ceremony* and what it means.
PK3	I understand that the vermilion dot symbolizes the enlightenment of wisdom.
PK4	I understand the meaning of “adjusting clothing and headgear.”
PK5	I understand the meaning of “drumming for wisdom.”
PK6	I understand the meaning of “writing the character ‘Ren’ (人).”
PA1	I found the gesture recognition in Pico accurate.
PA2	I found the gesture recognition in Leap Motion accurate.
PA3	I found the gesture recognition in Pico easy to learn.
PA4	I found the gesture recognition in Leap Motion easy to learn.
PA5	I found the gesture recognition in Pico easy to use.
PA6	I found the gesture recognition in Leap Motion easy to use.

## Abbreviations

VR: Virtual reality
HMD: Head-mounted display
DG: Design goal
SUS: System Usability Scale
NASA-TLX: NASA Task Load Index
